# Case report: A case of tetanus in a dog: cranial nerve involvement and imaging findings

**DOI:** 10.3389/fvets.2023.1271334

**Published:** 2023-11-06

**Authors:** Kylie Grant, Sam Long

**Affiliations:** Veterinary Referral Hospital, Melbourne, VIC, Australia

**Keywords:** tetanus, cranial nerve, vestibular disease, EMG, dog

## Abstract

An 11 years old male Labrador cross presented with unilateral vestibular signs, ipsilateral facial paresis, moderate obtundation, ptyalism, and paraparesis. MRI of the brain revealed diffuse, multifocal T2/FLAIR hyperintense changes throughout various regions of the brain including the medulla, midbrain, pons, thalamus and right cerebral hemisphere with mild multifocal contrast enhancement. The patient progressed to trismus with generalized increased extensor tone and risus sardonicus. A diagnosis of generalized tetanus was made and the patient was started on antibiotics, skeletal muscle relaxants and tetanus antitoxin and made a full recovery. To the best of the authors’ knowledge, this is the first reported case of canine tetanus in which the presenting signs involved cranial nerve dysfunction as well as the first report describing MRI changes in canine tetanus within the central nervous system.

## Introduction

The clinical syndrome of tetanus is caused by the exotoxin tetanospasmin, produced by the vegetative form of *Clostridium tetani*. The disease results from inoculation of the bacterial spores which subsequently germinate and elucidate toxin within animal tissue. The resulting toxin that is produced gains access to telodendrons of motor neurons at the neuromuscular junction either locally from a nearby wound or via haematogenous spread. The toxin is then able to travel via retrograde axonal transport to the central nervous system where it has a predilection for the inhibitory interneurons, particularly the Renshaw cells of the spinal cord. Within these cells, the toxin prevents the release of glycine and Gamma-Amino Butyric Acid (GABA) (1) thereby resulting in the clinical syndrome of muscle spasticity and rigidity. In addition to its effects on inhibitory neurotransmitters, tetanospasmin can affect the release of other neurotransmitters, including acetylcholine, and can affect sensory and autonomic nerve thereby causing both peripheral weakness and cranial nerve paresis as well as autonomic dysfunction (2, 3).

In people, tetanus presents as one of four forms: neonatal, localised, generalized, or cephalic (4). Cephalic tetanus is characterized by paresis of one or more cranial nerves, including cranial nerves III, IV, VI, VII, and XII and may present with symptoms including ptosis, facial palsy, nystagmus, dysarthria, facial spasm, and dysphagia in addition to trismus (5, 6). In addition, although MRI of the central nervous system of human tetanus patients is typically normal there have been sporadic case reports of changes within the brain and spinal cord associated with the condition (7, 8). This case report describes a case of generalized tetanus in a dog in which the clinical signs involved cranial nerve dysfunction, similar to cephalic tetanus in people. We also report MRI changes within the brain associated with the disease, similar to those reported in people.

## Case presentation

An 11 MN Labrador cross presented to the neurology service for a 3 weeks history of paraparesis. Previous bilateral tibial wedge levelling osteotomy procedures had been performed more than 12 months prior to presentation for bilateral cruciate ligament disease but no other significant medical history was noted and no recent surgery or injuries had been recorded. For the 2 weeks prior to presentation the patient had also experienced difficulties prehending food and increased drooling. No dysphagia was noted by the owners.

Clinical examinations revealed pink mucous membranes, heart rate of 80 beats per minute, panting, rectal temperature 37.9 degrees Celcius, normal heart and chest sounds, comfortable on abdominal palpation and peripheral lymph nodes unremarkable on palpation. Neurological examination on initial presentation revealed mild vestibular ataxia and paraparesis with a tendency to circle to the left. Postural reaction deficits were present in both pelvic limbs with mild delayed paw replacement and markedly delayed lateral hopping in both pelvic limbs. Segmental reflexes were normal. Intermittent nystagmus with a fast phase to the right and mild left head tilt were noted together with reduced left menace response. Vision was normal bilaterally with reduced palpebral reflex on the left. The patient was moderately obtunded and significant ptyalism was observed. Neurolocalisation was consistent with multifocal disease involving the caudal fossa (with cranial nerves VII and VIII involved) and T3-L3 spinal cord.

Blood was taken for haematology and biochemistry panels, which revealed a mild neutrophilia and monocytosis, mild hyperphosphataemia and mild increased ALT (Table 1). TT4 was also assessed and reported to be within normal reference ranges.

**Table 1:** Haematology and biochemistry results.

Test	Result	Reference range
Red Cell Count × 10^12^/L	5.50	5.46–8.39
HctL/L	0.40	0.37–0.55
Hgb g/L	131	130–200
MCVfl	72	61–77
MCHpg	24	21–26
MCHC g/L	330	324–363
Platelet × 10^9^/L	292	200–500
White Cell Count × 10^9^/L	14.7	5.3–14.9
Segmented Neutrophil × 10^9^/L	12.0	3.1–10.3
Band Neu × 10^9^/L	0	0–0.2
Lymphocyte × 10^9^/L	1.3	0.9–4.1
Monocyte × 10^9^/L	1.1	0.2–1.0
Eosinophil × 10^9^/L	0.2	0–1.3
Basophil × 10^9^/L	0	0–0.1
Total Protein g/L	59	53–73
Albumin g/L	28	27–38
Globulin g/L	31	23–38
Bicarbonate mmol/L	22	17–26
Glucose mmol/L	4.5	3.4–6.7
Na mmol/L	148	142–154
K mmol/L	4.6	4.0–5.8
Cl mmol/L	112	103–116
Anion gap mmol/L	19	12–26
Ca mmol/L	2.26	1.7–2.7
Phosphate mmol/L	2.12	0.8–1.9
Urea mmol/L	4.0	9.2
Creatinine umol/L	61	39–117
Total bilirubin umol/L	2	0–4
ALT IU/L	422	13–98
AST IU/L	64	17–84
ALP IU/L	171	0–182
CK IU/L	272	73–522
Cholesterol mmol/L	5.98	3.3–10.0
Amylase IU/L	675	334–1,277
Lipase IU/L	43	0–75
Total T4 nmol/L	25	13–47

**Figure 1:**
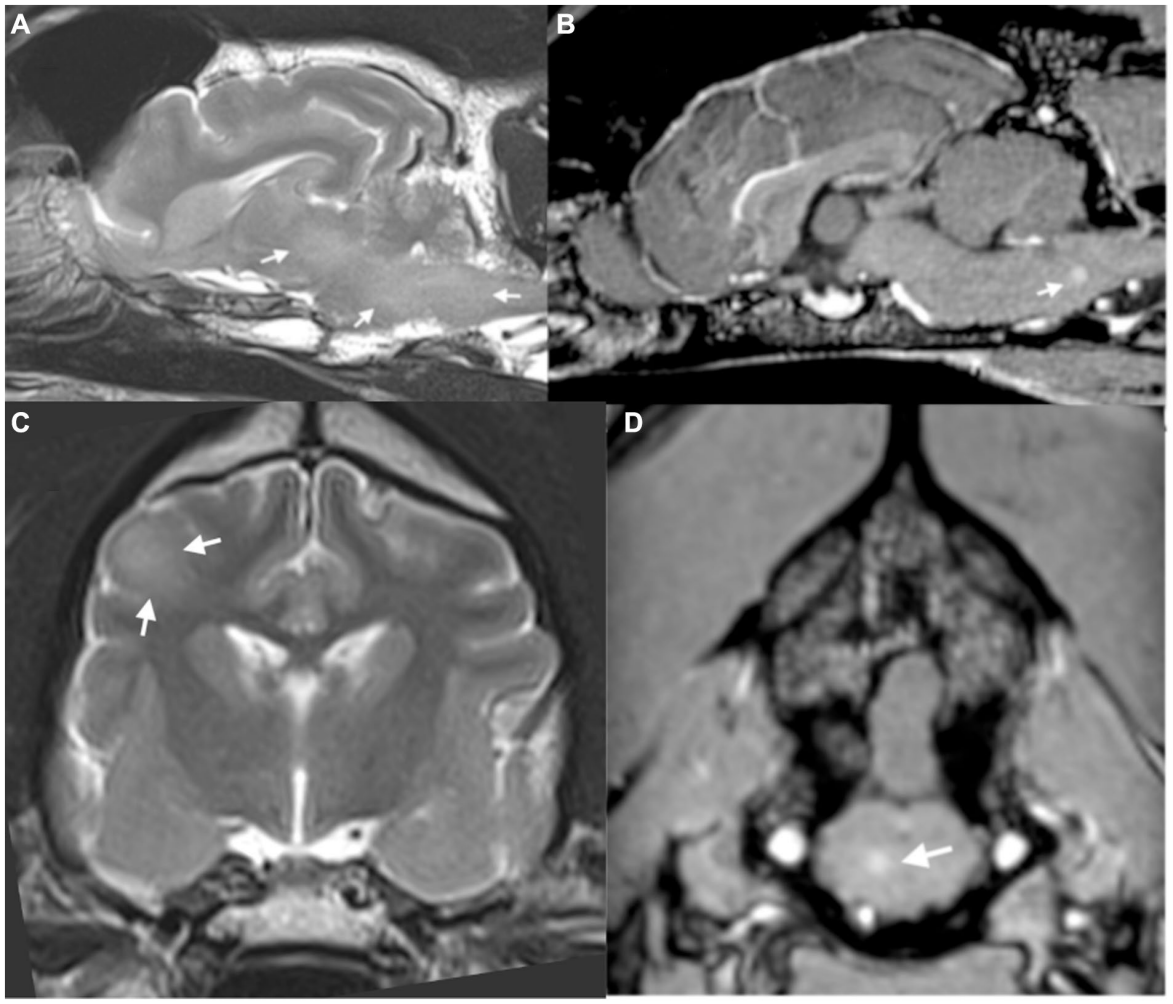
**(A)** Parasagittal T2-weighted image of the brain, showing diffuse T2-weighted hyperintensity within the brainstem (arrows). **(B)** Sagittal plane post-contrast T1-weighted image showing focus of contrast enhancement within the medulla (arrows). **(C)** Transverse plane T2-weighted image of the brain at the level of the interthalamic adhesion showing diffuse T2-weighted hyperintensity within the right parietal lobe (arrows). **(D)** Transverse post-contrast T1-weighted image at the level of the caudal cerebellar vermis showing small focus of contrast enhancement within the brainstem (arrow).

MRI of the brain and thoracolumbar spine was performed (3 T Siemens Magnetom Skyra, Erlangen, Germany). MRI of the brain revealed diffuse, patchy, multifocal, subtle hyperintensities on T2 weighted images within the medulla, the pons, the midbrain and thalamus (Figure 1A), as well as within the corona radiata of the right cerebral hemisphere in the region of the parietal lobe (Figure 1C). Mild multifocal contrast enhancement was noted within the internal capsule, corona radiata, the medial thalamic nuclei, the midbrain and the periphery of the pons and medulla (Figures 1B,D). Multiple dehydrated intervertebral disks throughout the thoracic and lumbar spinal cord but no significant compression was noted along the length of the spinal cord that was imaged. The intracranial findings were consistent with inflammatory CNS disease including non-infectious inflammatory disease such as meningoencephalitis or unknown origin, infectious inflammatory disease such as viral, bacterial, protozoal, fungal and amoebic encephalitis and multifocal/diffuse neoplasia such as lymphoma. A secondary T3-L3 myelopathy was considered likely given the presence of proprioceptive deficits in the pelvic limbs and normal proprioception and gait in the thoracic limbs at this stage, although no structural changes were found within the thoracolumbar spinal cord to adequately explain this.

The patient returned the following week for cerebrospinal fluid collection. No treatment was given in the interim to avoid interfering with possible treatment options. On representation he was obtunded with obvious difficulty opening the mouth, along with ongoing ptyalism with episodes of jaw chattering. Marked increased extensor tone was noted in all limbs. His ears were drawn back on both sides and enophthalmos with bilateral third eyelid protrusion and narrowing of the palpebral fissures were noted. Ongoing mild left head tilt was present, together with a left ventrolateral strabismus and reduced oculocephalic reflex. These findings were consistent with more extensive multifocal central nervous system disease involving the caudal brainstem and spinal cord.

CSF was collected via cerebellomedullary cistern puncture and submitted for analysis, which revealed mild albuminocytological dissociation but no other abnormalities (Table 2). An infectious disease PCR panel was performed on CSF for toxoplasma, cryptococcus, angiostrongylus and neospora DNA and canine distemper virus RNA, all of which were negative. CSF was also submitted for antibody titre testing for neospora which was negative. Latex cryptococcal antigen titres (LCAT) were also analysed and were negative.

**Table 2:** CSF tap results (cerebellomedullary cistern sample).

Test	Result	Reference interval	Unit
TNCC	0	<5	Cells/uL
RCC	0	0	Cell/uL
TP	0.41	<0.30	g/L

**Figure 2:**
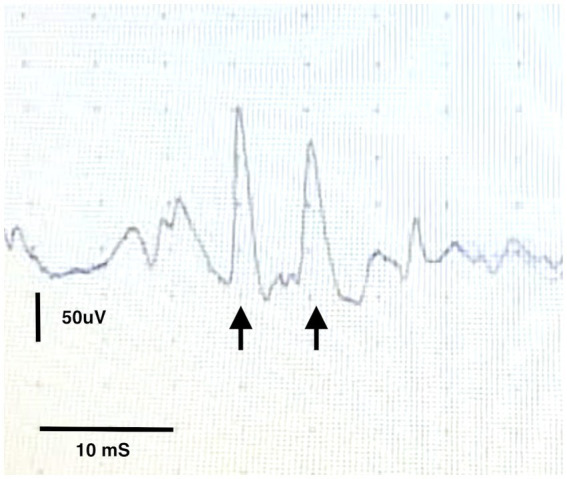
Conscious EMG from the temporalis muscle showing a motor unit potential firing twice within 20 ms (arrows).

On recovery from general anaesthesia the patient was noted to have worsened trismus and obvious risus sardonicus. A conscious concentric needle EMG examination of the periauricular muscles was performed with the animal immobile which revealed marked high amplitude spontaneous activity with frequent doublets (Figure 2). An extensive search for a penetrating wound or injury was performed, with particular attention paid to interdigital webbing, the oral mucosa, and the pharynx and larynx. No obvious injury was identified.

At this point, based on the combination of clinical signs and EMG findings, a diagnosis of generalised tetanus was made. Tetanus antitoxin was administered as a slow intravenous bolus over 30 min after 0.1 mL was administered subcutaneously to assess for anaphylaxis (Equivac TAT, Zoetis) and the patient was started on skeletal muscle relaxants (diazepam: 0.5 mg/kg q8 and dantrolene 1.5 mg/kg q12 PO). An oesophagostomy tube was placed to allow adequate nutrition and medication administration. Antibiotics (amoxycillin-clavulanic acid at 15 mg/kg intravenously q8 and then transitioned to oesophagostomy tube administration after 48 h) were also commenced. Recumbent patient care was administered with 4 hourly rotation, bladder expression, oral and ocular care and oesophagostomy tube feeding throughout hospitalisation.

The patient was hospitalised for 7 days and over the course of his hospitalisation gradual improvement was noted in his mental status, extensor tone of his appendicular and facial muscles and improvement in trismus. At discharge the patient was ambulatory with assistance with moderate increased appendicular extensor tone, eating without assistance, resolution of his head tilt, strabismus and physiological nystagmus. Reassessment 5 weeks post discharge revealed a normal neurological examination although a mild stiff gait remained.

## Discussion

To the best of the authors’ knowledge, this is the first reported case of canine tetanus in which the presenting signs involved cranial nerve dysfunction, similar to cephalic tetanus in people. Cephalic tetanus is a rare entity in people, with only 1–3% of all cases of tetanus presenting in this fashion (5). Most commonly the facial nerve is affected, with the condition being a commonly overlooked differential for Bell’s Palsy (9). Vestibular signs, however are less common, with only 1 case report describing bilateral vestibulopathy in a 58 years old male (10). The aetiology of cranial nerve involvement is poorly understood – it is unclear whether large amounts of tetanus toxin may interfere with presynaptic release of acetylcholine at the neuromuscular junction of cranial nerves or whether the site of dysfunction occurs at the cell body (10). Tetanospasmin prevents the release of presynaptic neurotransmitter for several weeks at least, and possibly permanently, with recovery likely to involve the formation of new synapses (4). *Clostridium tetani* also produces two other toxins – tetanolysin, and nonspasmogenic toxin. Tetanolysin causes haemolysis of red blood cells and is thought to aid the penetration of the toxin but does not contribute to clinical disease (1). The actions of nonspasmogenic toxin are less well understood but it is thought to cause peripheral nerve paralysis via neuromuscular blockage (1).

Germination of the spores of *C. tetani* requires anaerobic conditions. Commonly, cases of tetanus are reported following deep penetrating injuries, although surgical wounds have also been reported to provide suitable conditions for germination (11, 12). Treatment of the disease therefore involves debridement and cleaning of the initiating wound, if identifiable, as well as systemic antibiotic administration (13). Despite extensive searching we were unable to identify an obvious injury or wound which was the originating source of this dog’s infection. However, it is not uncommon for cases to present without an obvious wound (13). Given the chronicity of the development of this dog’s signs, it is possible that the inciting lesion had already healed by the time of presentation, although this cannot be confirmed. Mortality rates of up to 50% in canines have been reported in some case series (14, 15), and recently it has been suggested that the use of magnesium can improve survival, although it was not used in this case (16).

To the authors’ knowledge this is also the first case report of MRI changes within the central nervous system associated with tetanus in dogs. The imaging findings, combined with an elevated protein count on CSF are consistent with inflammatory changes within the brain. Although a concurrent inflammatory/infectious encephalopathy cannot be completely excluded our diagnostic testing did not identify any other putative viral cause. Given the lack of meningeal involvement on MRI and the fact that the lesions present were largely within the deep neuropil it is not unsurprising that a lack of pleocytosis was noted with increased protein levels. Although MRI of the central nervous system of human tetanus patients is typically normal there have been sporadic case reports of changes within the brain and spinal cord associated with the condition (7, 8).

The first pertains to a 47 yr. old woman with severe generalised tetanus with hypo- and hyperintense lesions on T1W and T2W images, respectively, with contrast enhancement within the right parietal and frontal cortex and subcortical regions with cerebral atrophy (7). These lesions were theorised to be secondary to disruption of cortical GABAregic inhibitory pathways which are postulated to exist in humans. The second case relates to a 35 yr. male with focal tetanus and mild focal T2W hyperintensity within the thoracic spinal cord from the 7th to the 9th thoracic vertebra which was grey matter-centric (8). The MRI changes in our patient may reflect toxin impairment of brainstem and higher cortical inhibitory pathways resulting in increased neuronal excitation and evidence of suspected inflammatory changes. This theory seems plausible given that children who have survived neonatal tetanus infections have been documented to suffer from brain damage such as developmental impairment and microcephaly (17).

Ultimately, our diagnosis of generalised tetanus was based on characteristic clinical findings of appendicular muscle extensor rigidity, risus sardonicus and trismus as well as consistent EMG findings. One of the limitations of our case is the diagnosis of tetanus being made on stereotypical clinical signs and response to treatment. A repeat MRI of the brain to assess for resolution of the reported MRI changes was unfortunately not feasible due to owner concerns and financial limitations. Interestingly in the case of the 47 years old woman previously mentioned, MRI changes were apparent on days 82, 95 and 185 post-hospitalisation, leaving the question of when and if these changes resolve. Commonly the diagnosis of tetanus in dogs is made with the help of the stereotypical clinical signs – the appearance of the ‘sawhorse’ stance and the appearance of the face as a result of extreme muscle extension are to be found in many textbooks. For this reason, atypical clinical signs and progression pose a diagnostic challenge to the clinician. Culture of *clostridium tetani* can be performed if a likely wound can be identified. However, this is challenging and often unrewarding and no wound or source of inoculation of the bacterium was identified in this animal. Measurement of serum tetanus antibody titres against tetanus toxin can also be performed and compared with control animals however this test is difficult to facilitate and a negative test result may reflect a low level of tetanospasmin insufficient to produce a detectable immune response. For this reason, antibody titres are rarely performed in dogs. EMG changes have previously been reported to aid in the diagnosis of canine and feline localized tetanus (18, 19). Specifically, the appearance of doublets, although not pathognomonic for the condition, was consistent with the diagnosis of tetanus. Doublets and triplets describe EMG patterns in which a single motor unit fires twice or three times in quick succession – the action potentials arising from this motor unit appear identical and with a fixed relationship to one another with an interval between them of between 2 and 20 ms (20). The term “paired discharges” has also been used to refer to similar motor unit repetitive firing but with an interval of 20 to 80 ms between successive waveforms. Doublets, triplets and paired discharges are commonly seen with diseases that are associated with hyperexcitability of the motoneuron pool – in addition to tetany, they may accompany cramps, hyperventilation and other metabolic states [examples of associated disease states include poliomyelitis, motor neuron disease, Guillain Barre syndrome, radiculopathy and myotonic dystrophy (20)]. Whilst repeating the EMG under general anaesthesia would have been ideal to demonstrate the persistence of these abnormal spontaneous discharges to rule out interference from voluntary muscle contractions, we did not feel it necessary at that stage to corroborate our suspicions of tetanus given the presence by this point of the more classic clinical signs of tetanus. Additionally, EMG was conducted in a quiet environment and the presence of doublets is suggestive of hyperexcitability rather than voluntary muscle contraction.

## Conclusion

We have described the first reported case of tetanus in the dog in which the initial presenting signs related to cranial nerve involvement. In addition, this is the first report of MRI changes identified within the brain of a patient suffering from tetanus. The combination of conscious EMG abnormalities and progression of clinical signs ultimately allowed a diagnosis to be made but this case serves to warn the clinician that tetanus should be included in the list of differential diagnoses for patients presenting with signs of cranial nerve dysfunction and caudal fossa disease alongside the more classic signs of generalized tetanus.

## Data availability statement

The raw data supporting the conclusions of this article will be made available by the authors, without undue reservation.

## Author contributions

KG: Writing – original draft. SL: Writing – review & editing.

## References

[1] Greene C. Infectious diseases of the dog and cat. 4th. St Louis: Elsevier. (2012). 423–431

[2] Humeau Y Doussau F Grant NJ. How botulism and tetanus neurotoxins block neurotransmitter release. Biochimie. (2000) 82:427–46. doi: 10.1016/S0300-9084(00)00216-9, PMID: 10865130

[3] Hassel B. Tetanus: pathophysiology, treatment, and the possibility of using botulinum toxin against tetanus-induced rigidity and spasms. Toxins. (2013) 5:73–83. doi: 10.3390/toxins5010073, PMID: 23299659 PMC3564069

[4] Bleck TP. Tetanus: pathophysiology, management, and prophylaxis. Dis Mon. (1991) 37:545–603. doi: 10.1016/0011-5029(91)90008-Y, PMID: 1874121

[5] Jagoda A Riggio S Burguieres T. Cephalic tetanus: a case report and review of the literature. Am J Emerg Med. (1988) 6:128–30. doi: 10.1016/0735-6757(88)90049-6, PMID: 3281682

[6] Burgess JA Wambaugh GW Koczarski MJ. Report of case: reviewing cephalic tetanus. J Am Dent Assoc. (1992) 123:67–70. doi: 10.14219/jada.archive.1992.0170 1619169

[7] Ijichi T Yamada T Yoneda S Kajita Y Nakajima K Nakagawa M. Brain lesions in the course of generalised tetanus. J Neurol Neurosurg Psychiatr. (2003) 74:1432–4. doi: 10.1136/jnnp.74.10.1432, PMID: 14570841 PMC1757387

[8] Tsuchiya M Kobayashi F Yamashiro N Nagasaka T Shindo K Takiyama Y. A case of local tetanus presenting spastic paraplegia mimicking myelitis. Rinsho Shinkeigaku. (2018) 58:688–91. doi: 10.5692/clinicalneurol.cn-001196, PMID: 30369524

[9] Kotani Y Kubo K Otsu S Tsujimoto T. Cephalic tetanus as a differential diagnosis of facial nerve palsy. BMJ Case Rep. (2017) 2017:bcr2016216440. doi: 10.1136/bcr-2016-216440, PMID: 28108438 PMC5255546

[10] Kagoya R Iwasaki S Chihara Y Ushio M Tsuji S Murofushi T. Cephalic tetanus presenting as acute vertigo with bilateral vestibulopathy. Acta Otolaryngol. (2011) 131:334–6. doi: 10.3109/00016489.2010.526144, PMID: 21133652

[11] Rubin S Faulkner RT Ward GE. Tetanus following ovariohysterectomy in a dog: a case report and review. J Am Anim Hosp Assoc. (1983) 19:293–8.

[12] Bagley RS Dougherty SA Randolph JF. Tetanus subsequent to ovariohysterectomy in a dog. Prog Vet Neurol. (1994) 5:63–5.

[13] Burkitt J Sturges B Jandrey K Hass P. Risk factors associated with outcome in dogs with tetanus: 38 cases (1987–2005). JAVMA. (2007) 230:76–83. doi: 10.2460/javma.230.1.76 17199496

[14] Adamantos S Boag A. Thirteen cases of tetanus in dogs. Vet Rec. (2007) 161:298–302. doi: 10.1136/vr.161.9.298, PMID: 17766808

[15] Bandt C Rozanski E Steinberg T Shaw S. Retrospective study of tetanus in 20 dogs: 1988–2004. JAAHA. (2007) 43:143–8. doi: 10.5326/0430143 17473020

[16] Papageorgiou V Kazakos G Anagnostou T. Polizopoulou, the role of magnesium in the management of acute and long-term symptoms caused by tetanus in two dogs. Topic Compan Anim Med. (2021) 44:100535. doi: 10.1016/j.tcam.2021.100535, PMID: 33933700

[17] Barlow JL Mung’Ala-Odera V Gona J Newton CRJC. Brain damage after neonatal tetanus in a rural Kenyan hospital. Tropical Med Int Health. (2001) 6:305–8. doi: 10.1046/j.1365-3156.2001.00705.x, PMID: 11348521

[18] Bianchi E Biserni R Gallucci A Pisoni L Menchetti M Gandini G. Changes in electromyography and F wave response in two cats with presumed local tetanus: implications for diagnosis and prognosis. J Feline Med Surg. (2013) 15:927–31. doi: 10.1177/1098612X13479599, PMID: 23439760 PMC11383150

[19] De Risio L Zavattiero S Venzi C Del Bue M Poncelet L. Focal canine tetanus: diagnostic value of electromyography. J Small Anim Pract. (2006) 47:278–80. doi: 10.1111/j.1748-5827.2006.00046.x, PMID: 16674723

[20] Kimura J. Electrodiagnostics in diagnosis of nerve and muscle principle and practice. 4th ed. New York: Oxford University Press (2013). 379 p.

